# Assessing the risks of children with preoperative comorbidities undergoing comminuted fracture surgery

**DOI:** 10.3389/fped.2023.1118954

**Published:** 2023-02-20

**Authors:** Kai-Yuan Cheng, Chen-Yu Yang, Shih-Chia Liu

**Affiliations:** Department of Orthopedics, MacKay Memorial Hospital, Taipei, Taiwan

**Keywords:** comorbidity, comminuted fracture, pediatrics, nationwide inpatient sample (NIS), trauma

## Abstract

**Introduction:**

Comminuted fractures are characterized by bones broken in at least two places, destabilizing the bone and requiring surgery. Children whose bones are still developing and maturing tend to have a higher risk of sustaining comminuted fractures as the result of trauma. Trauma is a major cause of death in children and constitutes a major issue in orthopedics because of the unique properties of children's bones compared to adult bones and the associated complications.

**Methods:**

This retrospective, cross-sectional study aimed to refine the association between comorbid disease and comminuted fractures in pediatric subjects using a large, national database. All data were extracted from the National Inpatient Sample (NIS) database from 2005 to 2018. Logistic regression analysis was used to evaluate associations between comorbidities and comminuted fracture surgery and between various comorbidities and LOS or unfavorable discharge.

**Results:**

A total of 2,356,483 patients diagnosed with comminuted fractures were selected initially, of whom 101,032 patients aged younger than 18 years who underwent surgery for comminuted fractures were included. Study results suggest that patients with any comorbidities undergoing orthopedic surgery for comminuted fracture appear to have longer LOS and a higher proportion of discharge to long-term care facilities.

**Discussion:**

Almost all comorbidities were significantly associated with poor in-hospital outcomes and longer LOS. The analysis of comminuted fractures in children may provide useful information to help first responders and medical personnel evaluate and manage comminuted fractures appropriately.

## Introduction

Children's bone structure is notably different from that in adults (1). Bone strength is a product of bone quality and mineralization and is largely determined by the maximum bone mass achieved during the growth years (2). The osteoid density of a child's bone is less than that of an adult's (3); that is, children's bones are softer than those of adults, causing them to bend rather than break transversely as in most adult fractures (4). Juvenile bone structure features Haversian canals, a network of minute canals that makes pediatric bones more porous than adult bones (5), helping to explain why they bend more easily than adult bones (6). Children's bones are surrounded by the periosteum, a thick layer of connective tissue that helps to protect bones from damage and fractures associated with trauma (7). The periosteum contains the bones' blood supply, which replaces damaged cells in fractures to aid in healing broken bones.

Bone fractures are classified as open or closed. A closed fracture occurs when the bone breaks, but the skin remains intact; open fractures, also called compound fractures, occur when a broken bone penetrates the skin (8). Fractures occur when more force is applied to a bone than the bone can withstand, and the amount and type of force affects the type of fracture and the difficulty of repairing bone structure. The developing bones of children as well as their unique structure are responsible for the high frequency of comminuted fractures in children compared to the frequency in adults (9). External fixation modalities such as splints and casts are often insufficient to treat this type of fracture, and infection is a common complication of open fractures (10).

Comminuted fractures occur when a bone is under extreme stress or receives a high-force impact sufficient to cause multiple disruption in bone structure continuity (11). Fractures of this magnitude occur after high-impact trauma, such as in sports injuries or vehicle accidents, due to the considerable force required to break the bone (12). The impact force is the leading factor in determining the severity of comminuted fractures, but bone health still plays a role in preventing fractures (13).

Comminuted fractures typically require open surgery to rebuild the bone according to its normal anatomy. Reconstruction of these fractures can be relatively difficult, requiring careful treatment planning to achieve reliable functional outcomes with minimal complications (14, 15). Internal fixation has the advantages of stable fixation and accurate reduction, but because reduction requires extensive soft tissue dissection, risk of infection is especially high.

Children's bones are still developing and maturing, and many other factors such as nutrition, growth patterns and levels of activity may contribute to weak bones that tend to have a higher risk of sustaining comminuted fractures (16, 17). The management of comminuted fractures in pediatrics is a major orthopedic challenge because of the high incidence of complications and poor outcomes associated with these fractures (18). In recent years, researchers have paid more attention to the short- and long-term effects of childhood diseases on bone health. However, specific subgroup analyses and data of comminuted fractures in children are scarce. Therefore, this study aimed to evaluate the association between comorbid diseases and risk of comminuted fracture in pediatric subjects using a large nationwide database.

## Methods

### Study design and data source

This population-based, retrospective observational study extracted all data from the US Nationwide Inpatient Sample (NIS) database, which is the largest all-payer, continuous inpatient care database in the United States, including about 8 million hospital stays each year (19). The database is administered by the Healthcare Cost and Utilization Project (HCUP) (“ahrq.gov/data/hcup/index.html” http://ahrq.gov/data/hcup/index.html) of the US National Institutes of Health (NIH). Patient data include primary and secondary diagnoses, primary and secondary procedures, admission and discharge status, patient demographics, expected payment source, duration of hospital stay, and hospital characteristics (i.e., bed size/location/teaching status/hospital region). All admitted patients are initially considered for inclusion. The continuous, annually updated NIS database derives patient data from about 1,050 hospitals from 44 States in the US, representing a 20% stratified sample of US community hospitals as defined by the American Hospital Association.

### Ethics statement

All data were obtained through request to the Online Healthcare Cost and Utilization Project (HCUP) Central Distributor, which administers the database (certificate #HCUP-29L41EYS3). This study conforms to the NIS data-use agreement with HCUP. Because this study analyzed secondary data from the NIS database, patients and the public were not involved directly. The study protocol was submitted to the Institutional Review Board (IRB) of MacKay Memorial Hospital, which exempted the study from IRB approval. Since all data in the NIS database are de-identified, the requirement for informed consent was also waived.

### Study population

The data of pediatric patients aged under 18 years who were admitted to US hospitals with comminuted fracture and who received subsequent surgery were included as the analytic sample. Diagnoses of all patients and types of surgery (reduction or fixation) were confirmed usingthe International Classification of Diseases, Ninth and Tenth edition (ICD-9 and ICD-10) diagnostic codes the subjects without data of the main study endpoints were excluded.

### Study variables and outcome measures

The in-hospital outcomes, including length of stay (LOS), unfavorable discharge, and morbidities, were compared between patients with or without comorbidities.

### Covariates

Patients' demographic characteristics included age, race, and insurance status. Clinical characteristics included major comorbidities that were identified using the same ICD coding system. Comorbidities included in this analysis were diabetes, developmental delays, hypertension, dyslipidemia, obesity, seizures, coagulopathy, asthma, and any malignancy. Hospital-related characteristics such as bed size, location/teaching status, and hospital region were extracted from the database as part of the comprehensive data available for all participants.

### Statistical analysis

Considering the complex sampling design of HCUP-NIS data, all analyses were performed using SAS survey analysis statements (version 9.4, SAS Institute Inc., Cary, NC, United States). The NIS database includes sample sets that cover 20% of annual inpatient admissions in the United States. Weighted samples were used (TRENDWT used before 2011 and DISCWT after 2012), stratum (NIS_STRATUM), and cluster (HOSPID) to produce national estimates for all analyses. Continuous variables are presented as weighted means and standard error; categorical variables are presented as weighted numbers and weighted proportions. Differences in means between groups were compared using SURVEYREG procedure for continuous variables, while Rao–Scott *χ*^2^ test was performed to examine differences in the proportions between groups using SURVEYFREQ procedure for categorical variables. Linear regression was performed using SURVEYREG procedure to evaluate the beta coefficient (β) and 95% confidence interval (CI) between comorbidities and hospital length of stay (LOS). Logistic regression was performed using SURVEYLOGISTIC statement to evaluate the odds ratios (ORs) and 95% CIs of associations between comorbidities and unfavorable discharge and between morbidities and unfavorable discharge. Variables that reached statistical significance in univariate analysis were entered into multivariate models and confounding variables were adjusted. A two-sided *p*-value of <0.05 was regarded as statistical significance.

## Results

### Study population

**Figure 1:**
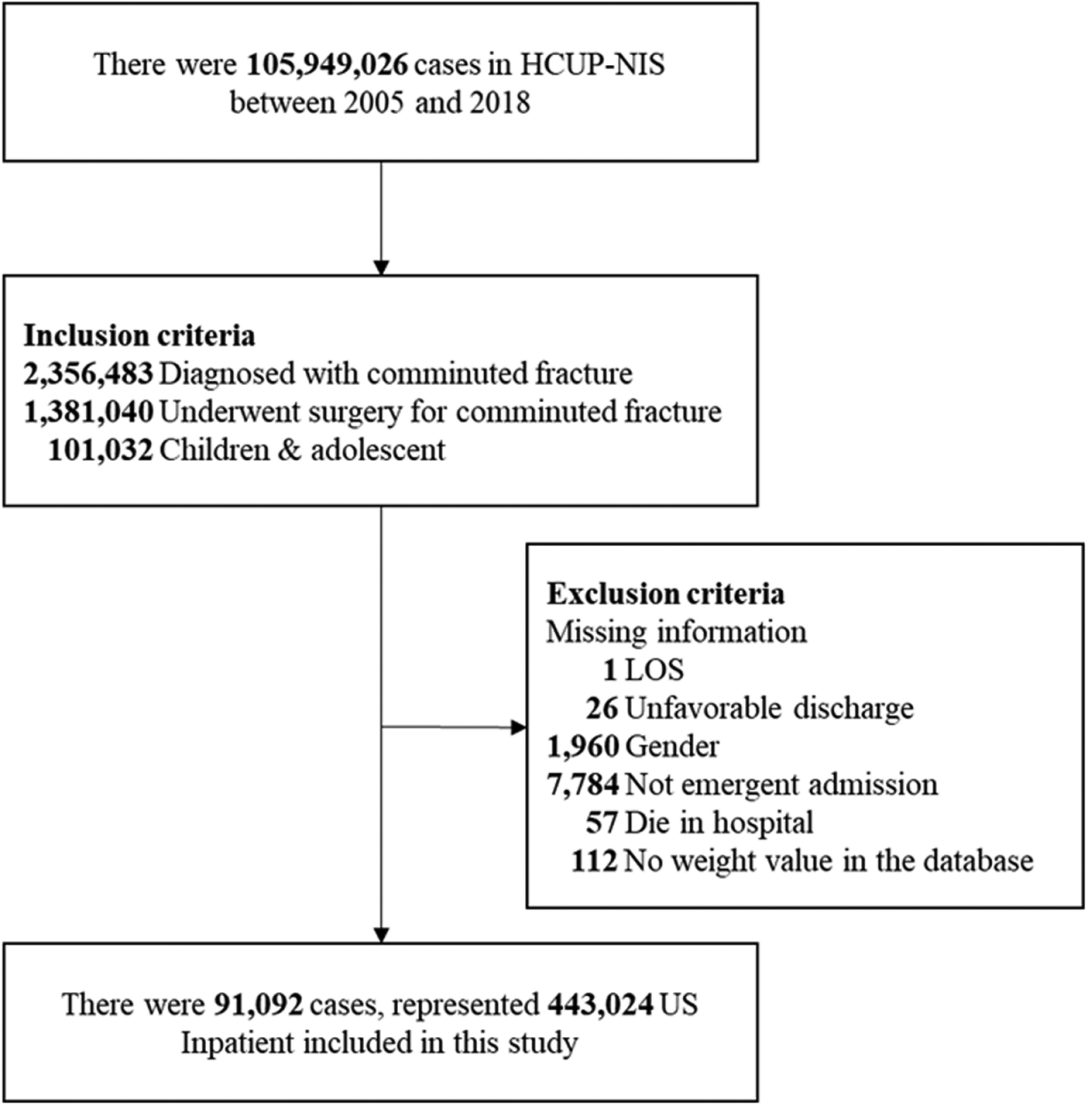
Flow diagram of study sample selection.

All data were extracted from the HCUP-NIS 2005–2018 database. A total of 2,356,483 patients with the diagnosis of comminuted fracture were selected. Among them, 101,032 patients aged younger than 18 years who underwent surgery for comminuted fracture were included. Patients with any missing information on LOS, unfavorable discharge, age, gender, elective status, in-hospital death, and weight were excluded. After exclusions, data of the remaining 91,092 patients were included in the analysis (Figure 1).

### Demographic and clinical characteristics of the study population

The mean age of included patients was 9.8 ± 0.05 years, and males accounted for 68%. More than half of the patients underwent the surgery with fixation (59.5%) and with private or health maintenance organization (HMO) insurance status (53.6%). The mean LOS was 2.6 ± 0.03 days. In addition, only a low percentage of patients were discharged to long-term care facilities (1.9%) compared to those with other unfavorable discharge. The most common postoperative morbidity among the patients was mechanical ventilation (1.6%) (Table 1).

**Table 1:** Demographic and clinical characteristics of the study population.

Variables	Overall *n* = 91,092	No comorbidities *n* = 80,731 (88.6%)	With comorbidities *n* = 10,361 (11.4%)	*p*-value
**LOS**	2.6 ± 0.03	2.5 ± 0.02	3.8 ± 0.07	**<0**.**001**
**Unfavorable discharge**				**<0**.**001**
Home/routine/short-term hospital (ref)	89,399 (98.1)	79,477 (98.4)	9,922 (95.7)	** **
Long-term care facility	1,693 (1.9)	1,254 (1.6)	439 (4.3)	** **
**Morbidity (postoperative)**				
AMI	2 (0.002)	1 (0.001)	1 (0.01)	0.086
CVA	46 (0.05)	36 (0.05)	10 (0.1)	**0**.**027**
VTE	174 (0.2)	126 (0.2)	48 (0.5)	**<0**.**001**
Pneumonia	258 (0.3)	191 (0.2)	67 (0.6)	**<0**.**001**
Sepsis	480 (0.5)	340 (0.4)	140 (1.3)	**<0**.**001**
Infection	783 (0.9)	573 (0.7)	210 (2.0)	**<0**.**001**
Respiratory failure	1,121 (1.2)	756 (0.9)	365 (3.5)	**<0**.**001**
Mechanical ventilation	1,437 (1.6)	1,046 (1.3)	391 (3.8)	**<0**.**001**
Aute kidney injury	98 (0.1)	49 (0.1)	49 (0.5)	**<0**.**001**
**Age**	9.8 ± 0.05	9.6 ± 0.05	10.9 ± 0.06	**<0**.**001**
0–5	22,173 (24.4)	20,428 (25.4)	1,745 (16.9)	**<0**.**001**
6–11	28,060 (30.8)	25,024 (31.0)	3,036 (29.4)	** **
12–17	40,859 (44.8)	35,279 (43.6)	5,580 (53.8)	** **
**Gender**				**<0**.**001**
Male	61,929 (68.0)	54,416 (67.4)	7,513 (72.5)	** **
Female	29,163 (32.0)	26,315 (32.6)	2,848 (27.5)	** **
**Type of surgery**				**0**.**048**
Reduction alone	36,617 (40.5)	32,357 (40.4)	4,260 (41.5)	** **
With fixation	54,457 (59.5)	48,361 (59.6)	6,096 (58.5)	
Missing	18	13	5	
**Surgical site**				**<0**.**001**
Reduction (Upper limb)	16,588 (18.5)	14,918 (18.7)	1,670 (16.4)	** **
Fixation (Upper limb)	29,322 (32.2)	26,695 (33.1)	2,627 (25.4)	
Reduction (Lower limb)	19,812 (22.0)	17,255 (21.7)	2,557 (25.0)	
Fixation (lower limb)	24,795 (27.3)	21,351 (26.5)	3,444 (33.2)	
Missing	575	512	63	
**Insurance status/Primary Payer**				**<0**.**001**
Medicare/Medicaid	33,139 (36.5)	28,699 (35.7)	4,440 (43.0)	** **
Private including HMO	48,638 (53.6)	43,548 (54.1)	5,090 (49.2)	
Self-pay/no-charge/other	9,075 (9.9)	8,270 (10.2)	805 (7.7)	
Missing	240	214	26	
**Household income**				**<0**.**001**
Q1	25,316 (28.4)	22,113 (28.0)	3,203 (31.8)	** **
Q2	21,855 (24.5)	19,361 (24.5)	2,494 (24.7)	
Q3	20,813 (23.3)	18,556 (23.5)	2,257 (22.3)	
Q4	21,053 (23.7)	18,925 (24.0)	2,128 (21.2)	
Missing	2,055	1,776	279	
**Hospital region**				**<0**.**001**
Northeast	18,156 (20.3)	15,795 (19.9)	2,361 (23.2)	** **
Midwest	17,417 (19.2)	15,504 (19.3)	1,913 (18.5)	
South	33,688 (36.5)	29,940 (36.6)	3,748 (35.8)	
West	21,831 (24.0)	19,492 (24.2)	2,339 (22.5)	
**Weekend admission**				**0**.**001**
No	60,755 (66.7)	53,708 (66.5)	7,047 (68.1)	** **
Yes	30,337 (33.3)	27,023 (33.5)	3,314 (31.9)	
**Hospital bed size**				0.111
Small	10,469 (11.2)	9,320 (11.2)	1,149 (10.8)	
Medium	20,537 (22.9)	18,274 (23.0)	2,263 (22.1)	
Large	59,588 (65.9)	52,696 (65.8)	6,892 (67.1)	
Missing	498	441	57	
**Hospital location/teaching status**				**<0**.**001**
Rural	5,737 (6.3)	5,185 (6.4)	552 (5.3)	** **
Urban nonteaching	17,865 (19.3)	16,073 (19.6)	1,792 (17.1)	
Urban teaching	66,992 (74.3)	59,032 (73.9)	7,960 (77.6)	

Categorical variables are presented as unweighted counts (weighted percentage) and continuous variables are presented as mean ± standard error (S.E.).

Significant values are shown in bold.

LOS, length of hospital stay; AMI, acute myocardial infarction; CVA, cerebrovascular accident; VTE, venous thromboembolism; HMO, health maintenance organization.

A total of 10,361 patients had at least one comorbidity (11.4%). Patients with or without comorbidities showed significant differences in LOS, unfavorable discharge, several morbidities and all other covariates except for hospital bed size. Patients with any comorbidities seemed to have significantly longer LOS than those without comorbidities (mean: 3.8 days vs. 2.5 days, respectively), significantly higher proportions of discharge to long-term care facilities (4.3% vs. 1.6%, respectively) and all morbidities were significantly associated with comorbidities except for acute myocardial infarction (AMI). Also, patients with any comorbidities were older (mean: 10.9 years vs. 9.6 years), and had a higher proportion of males (72.5% vs. 67.4%, respectively) (Table 1).

In addition, major comorbidities were observed in the study population. The most common comorbidities were asthma (7.3%), obesity (1.8%), and seizures (1.0%) (Table 2). Subsequently, comorbidities with sample size >30 (diabetes, developmental delays, hypertension, dyslipidemia, obesity, seizures, coagulopathy, asthma, and any malignancy) were then considered to be dependent variables in both univariate and multivariate analyses (Table 2).

**Table 2:** Comorbidities.

Major comorbidities	Overall *n* = 91,092
Ischemic heart disease	7 (0.01)
Congestive heart failure	17 (0.02)
Atrial fibrillation	14 (0.02)
Diabetes	320 (0.4)
Developmental delays	741 (0.8)
Hypertension	633 (0.7)
Dyslipidemia	83 (0.1)
Obesity	1,592 (1.8)
Seizure	871 (1.0)
Other rheumatic disease	22 (0.02)
Coagulopathy	485 (0.5)
Asthma	6,649 (7.3)
Any malignancy	86 (0.1)

Categorical variables are presented as unweighted counts (weighted percentage).

### Association between comorbidities and LOS

Univariate analysis revealed that all comorbidities showed significant associations with LOS except for any malignancy. After adjusting for covariates that showed significance in univariate analysis, comorbidities, including developmental delays, dyslipidemia, and asthma, became not associated with LOS in multivariate analysis. Patients with coagulopathy had an additional 9.84 days (aβ: 9.84, 95% CI: 8.38–11.30) LOS compared to patients without coagulopathy (*p* < 0.001) (Table 3).

**Table 3:** Univariate and multivariate analysis of associations between comorbidities and LOS.

Variables	LOS			
	β (95% CI)	*p*-value	Adjusted β (95% CI)	*p*-value
**Diabetes**	**2.05** (**1.24, 2.87)**	**<0**.**001**	**1.13** (**0.30, 1.96)**	**0**.**008**
**Developmental delays**	**1.34** (**0.88, 1.79)**	**<0**.**001**	0.47 (−0.02, 0.97)	0.062
**Hypertension**	**5.75** (**4.67, 6.84)**	**<0**.**001**	**4.65** (**3.57, 5.74)**	**<0**.**001**
**Dyslipidemia**	**2.60** (**0.90, 4.30)**	**0**.**003**	0.45 (−1.19, 2.09)	0.590
**Obesity**	**1.44** (**1.18, 1.70)**	**<0**.**001**	**0.42** (**0.16, 0.67)**	**0**.**001**
**Seizure**	**2.95** (**2.25, 3.65)**	**<0**.**001**	**2.25** (**1.55, 2.96)**	**<0**.**001**
**Coagulopathy**	**10.80** (**9.32, 12.28)**	**<0**.**001**	**9.84** (**8.38, 11.30)**	**<0**.**001**
**Asthma**	**0.13** (**0.03, 0.23)**	**0**.**009**	−0.08 (−0.18, 0.01)	0.078
**Any malignancy**	0.75 (−0.27, 1.76)	0.152	0.06 (−1.08, 1.21)	0.915

Multivariate model was adjusted for age (category), type of surgery, surgical site, insurance status/primary payer, household income, hospital region, weekend admission, hospital bed size, hospital location/teaching status.

Significant values are shown in bold.

LOS, length of hospital stay.

### Associations between comorbidities and unfavorable discharge

Results of univariable regression analysis showed that all comorbidities except asthma were significantly associated with unfavorable discharge. In multivariable analysis, associations between covariates, including developmental delays and dyslipidemia, and unfavorable discharge became insignificant after adjusting for possible confounders. Also, patients with any comorbidities, including diabetes, hypertension, obesity, seizures, and coagulopathy, had significantly higher risks for discharge to long-term care facilities compared to those with no comorbidities (aOR: 1.90, 95% CI: 1.17–3.09; aOR: 2.33, 95% CI: 1.66–3.26; aOR: 2.07, 95% CI: 1.63–2.63; aOR: 3.59, 95% CI: 2.61–4.94; aOR: 13.44, 95% CI: 10.41–17.35, respectively) (Table 4).

**Table 4:** Univariate and multivariate analysis of associations between comorbidities and unfavorable discharge.

Variables	Unfavorable discharge (Long-term facility vs. Home/routine/short-term hospital)			
	OR (95% CI)	*p*-value	aOR (95% CI)	*p*-value
**Diabetes**	**4.09** (**2.67, 6.28)**	**<0**.**001**	**1.90** (**1.17, 3.09)**	**0**.**010**
**Developmental delays**	**2.21** (**1.53, 3.19)**	**<0**.**001**	1.45 (0.90, 2.35)	0.127
**Hypertension**	**4.72** (**3.55, 6.26)**	**<0**.**001**	**2.33** (**1.66, 3.26)**	**<0**.**001**
**Dyslipidemia**	**3.33** (**1.36, 8.15)**	**0**.**009**	0.90 (0.33, 2.45)	0.836
**Obesity**	**3.59** (**2.90, 4.45)**	**<0**.**001**	**2.07** (**1.63, 2.63)**	**<0**.**001**
**Seizure**	**4.38** (**3.39, 5.68)**	**<0**.**001**	**3.59** (**2.61, 4.94)**	**<0**.**001**
**Coagulopathy**	**20.66** (**16.65, 25.64)**	**<0**.**001**	**13.44** (**10.41, 17.35)**	**<0**.**001**
**Asthma**	1.04 (0.87, 1.23)	0.696	0.86 (0.71, 1.04)	0.123
**Any malignancy**	–	**–**	**–**	**–**

Multivariate model was adjusted for age (category), gender, surgical site, insurance status/primary payer, household income, hospital region, hospital bed size, hospital location/teaching status.

No patients with any malignancy were discharged to long-term facility.

Significant values are shown in bold.

### Associations between morbidities and comorbidities

Multiple models were constructed to evaluate associations between morbidities and comorbidities. Hypertension and coagulopathy were comorbidities associated with all of the morbidities, followed by developmental delays, obesity, and seizures. Developmental delays and seizures were significantly associated with these morbidities except for venous thromboembolism (VTE). In multivariate analyses, coagulopathy remained the strongest risk factor for all morbidities.

Moreover, patients with hypertension had 7.20 times the risk for sepsis compared with those without hypertension (aOR: 7.20, 95% CI: 4.79–10.84). Also, patients with any malignancy had 6.99 times the risk for infection compared with those with no malignancy (aOR: 6.99, 95% CI: 2.75–17.76) (Table 5).

**Table 5:** Univariate analysis and multivariate analysis of associations between comorbidities and morbidities.

Variables	VTE^a^	Pneumonia^b^	Sepsis^c^	Infection^d^	Respiratory failure^e^	Mechanical ventilation^f^
	OR (95% CI)	OR (95% CI)	OR (95% CI)	OR (95% CI)	OR (95% CI)	OR (95% CI)
**Univariate analysis**						
Diabetes	3.38 (0.83, 13.67)	**4.57** (**1.71, 12.27)**	1.80 (0.59, 5.53)	**3.23** (**1.66, 6.27)**	**2.40** (**1.24, 4.65)**	**3.07** (**1.83, 5.16)**
Developmental delays	0.72 (0.10, 5.13)	**5.03** (**2.67, 9.48)**	**4.60** (**2.85, 7.43)**	**5.70** (**4.02, 8.08)**	**3.78** (**2.65, 5.40)**	**2.51** (**1.72, 3.66)**
Hypertension	**8.05** (**4.15, 15.61)**	**5.71** (**3.05, 10.69)**	**11.80** (**8.27, 16.84)**	**7.37** (**5.26, 10.33)**	**9.79** (**7.39, 12.98)**	**8.64** (**6.72, 11.11)**
Dyslipidemia	**7.23** (**1.03, 50.86)**	3.99 (0.55, 28.83)	2.61 (0.36, 18.73)	1.60 (0.22, 11.47)	3.15 (0.99, 10.05)	1.51 (0.37, 6.12)
Obesity	**2.31** (**1.09, 4.88)**	**2.75** (**1.53, 4.93)**	**2.35** (**1.48, 3.72)**	**2.32** (**1.62, 3.32)**	**1.69** (**1.18, 2.41)**	0.79 (0.51, 1.24)
Seizure	1.24 (0.31, 5.01)	**4.77** (**2.61, 8.72)**	**6.85** (**4.67, 10.05)**	**5.83** (**4.22, 8.06)**	**6.62** (**5.10, 8.60)**	**8.88** (**7.16, 11.01)**
Coagulopathy	**20.81** (**12.59, 34.42)**	**10.09** (**5.74, 17.74)**	**11.33** (**7.37, 17.41)**	**8.58** (**5.97, 12.33)**	**40.57** (**32.77, 50.22)**	**26.83** (**21.69, 33.18)**
Asthma	1.18 (0.69, 2.00)	1.04 (0.66, 1.65)	**1.34** (**1.00, 1.80)**	**1.45** (**1.14, 1.83)**	0.97 (0.78, 1.22)	0.84 (0.68, 1.03)
Any malignancy	–	4.23 (0.58, 30.63)	**4.61** (**1.16, 18.37)**	**8.89** (**3.95, 20.03)**	–	0.68 (0.09, 4.89)
**Multivariate analysis**						
Diabetes	1.65 (0.36**–**7.62)	2.65 (0.91**–**7.69)	0.93 (0.29**–**3.03)	**2.15** (**1.04–4.44)**	1.19 (0.56**–**2.52)	1.78 (0.98**–**3.20)
Developmental delays	0.61 (0.08**–**4.97)	**3.12** (**1.49–6.51)**	**2.18** (**1.22–3.87)**	**3.12** (**2.06–4.73)**	**1.86** (**1.11–3.10)**	0.91 (0.54**–**1.53)
Hypertension	**3.55** (**1.58–7.99)**	**3.16** (**1.62–6.20)**	**7.20** (**4.79–10.84)**	**4.94** (**3.37–7.25)**	**5.67** (**3.92–8.21)**	**6.00** (**4.38–8.21)**
Dyslipidemia	2.58 (0.30**–**22.39)	1.17 (0.14**–**9.90)	0.71 (0.09**–**5.80)	0.43 (0.05**–**3.45)	1.07 (0.30**–**3.87)	0.55 (0.11**–**2.72)
Obesity	1.16 (0.52**–**2.58)	1.55 (0.81**–**2.96)	1.16 (0.70**–**1.95)	1.45 (0.99**–**2.14)	0.87 (0.57**–**1.35)	**0.41** (**0.24–0.68)**
Seizure	0.99 (0.22**–**4.44)	**2.68** (**1.31–5.47)**	**4.15** (**2.63–6.57)**	**3.29** (**2.25–4.79)**	**4.53** (**3.21–6.39)**	**7.11** (**5.43–9.30)**
Coagulopathy	**11.44** (**6.55–20.00)**	**6.69** (**3.75–11.94)**	**6.33** (**3.92–10.21)**	**5.81** (**3.92–8.60)**	**26.19** (**20.33–33.73)**	**18.11** (**14.12–23.23)**
Asthma	1.12 (0.66**–**1.89)	0.85 (0.52**–**1.38)	1.25 (0.92**–**1.69)	**1.38** (**1.08–1.76)**	0.87 (0.68**–**1.12)	**0.75** (**0.60–0.93)**
Any malignancy	–	2.78 (0.34**–**22.48)	3.16 (0.70–14.29)	**6.99** (**2.75–17.76)**	–	0.29 (0.04–2.29)

In the multivariate analysis, models were adjusted for:

^a^ Age (category), type of surgery, surgical site, hospital region, hospital location/teaching status.

^b^ Age (category), surgical site, household income, hospital region.

^c^ Age (category), sex, type of surgery, surgical site, insurance status/primary payer, household income, hospital region, hospital bed size, hospital location/teaching status.

^d^ Age (category), sex, type of surgery, surgical site, insurance status/primary payer, household income, hospital region, hospital location/teaching status.

^e^ Age (category), sex, type of surgery, surgical site, insurance status/primary payer, household income, hospital region, hospital bed size, hospital location/teaching status.

^f^ Age (category), sex, surgical site, insurance status/primary payer, household income, hospital region, hospital bed size, hospital location/teaching status.

Significant values are shown in bold.

VTE, venous thromboembolism.

## Discussion

The present study examined the impact of comorbidities on the risk profile of pediatric patients undergoing comminuted fracture surgery, finding that all comorbidities except asthma were significantly associated with poor discharge status. Except for dyslipidemia, asthma, and malignancy, patients with other comorbidities, including diabetes, developmental delays, hypertension, obesity, seizures, and coagulopathy tended to have worse in-hospital outcomes. The most common comorbidities were asthma, obesity, and seizures. The present study found that almost all comorbidities were significantly associated with LOS and poor discharge status. Although patients with obesity had a higher risk of unfavorable discharge, they tended to have shorter LOS.

Treatment for comminuted fractures in pediatric patients depends on the patient's symptoms, age, and general health as well as the location and severity of the fractures (20, 21). The ideal treatment for comminuted femoral fractures includes sufficient curettage and bone grafting, accurate reduction of the fractures and stable fixation—all without disturbing normal bone growth in pediatric patients (20–22). The pediatric physeal slide-traction plate is reported to be a safe and effective treatment for comminuted distal femoral fractures in children, primarily because it can be lengthened through reliable internal fixation as the epiphyseal plate continues to grow (22).

Previous studies have indicated that certain types of fractures, including nondisplaced, displaced, and comminuted fractures, tend to occur in specific anatomical sites in children, and the severity and extent of fractures vary according to the patient's age and stage of skeletal development (23, 24). Pediatric patients in the present study all had upper or lower limb fractures. Dependent on fracture location, pediatric patients will need to wear a splint or cast for a period of time after surgery to prevent the bones from moving as they heal.

Several authors have focused specifically on pediatric facial skeletal fractures that require specialized knowledge for diagnosis, treatment, and follow-up (23–25). In particular, Rogan and Ahmed (25) indicated that when treating children it is critical to understand the differences between the patterns of facial fractures in children vs. those in adults, which may also be true for fracture patterns elsewhere in the body.

The goal of surgical intervention for comminuted fractures is to restore bone length, alignment, and rotation (26). In comminuted or unstable fracture surgery, elastic intramedullary nails have been used commonly for many years, but they are shown to have uncertain control of rotation and length, and inadequate control of proximal or distal fractures in children (27). Nevertheless, another previous study indicated that the combination of an external fixator and an elastically stabilized intramedullary nail is a good method for treating comminuted long bone fractures in children (18), while other authors show intramedullary nails to be associated with complications and longer LOS (28). In the present study, more than half of the patients underwent internal fixation surgery, and patients with any comorbidities still had significantly longer LOS and a higher proportion were discharged to long-term care facilities.

Variations in the treatment of comminuted fractures have been reported. A previous study described an improvement over the traditional tension band structure that uses additional wires and multiple tension bands to collect and fix comminuted fracture patterns with satisfactory clinical outcomes (29). However, the extra wire and multiple tension bands may severely damage soft tissue surrounding the fracture, and several complications can occur, including wire displacement, K-wire breakage, pain from stainless steel wire loops, and protruding hardware. Another previous study indicated that some surgical treatment modalities carry the risk of limb length differences, deep infection, and refractures after plate removal (30).

Although many studies, including some of those cited above, have investigated surgery for comminuted fractures in children and in adults, we were not able to find investigations of relative risk in children with comorbid conditions undergoing comminuted fracture surgery. Therefore, we cannot easily compare results of the present study with previous results. A review of bone health in children with chronic disease has emphasized that children and adolescents with chronic diseases are predisposed to issues of bone health, including significant risk of demineralization and fractures (15). Lack of physical activity in these young people along with underlying inflammation and malabsorption of essential nutrients contribute to the increased risk. Other authors promote a systematic approach to trauma injuries in children, including comminuted fracture, emphasizing the unique anatomical properties of pediatric bone (1). Results of the present study are particularly timely in addressing both the initial risk of traumatic fracture in children and the additional risk posed by chronic disease in the management of this pediatric population.

## Limitations

The study is inherently limited by its retrospective and observational nature, which may limit the measurement of certain variables and selection bias cannot be ruled out. The possibility of coding errors also cannot be ruled out as in other studies that use ICD coding systems. This study also lacks data of clinical laboratory parameters and long-term follow-up data after patients are discharged, which are not provided in the NIS database.

## Conclusions

All comorbidities in pediatric patients are associated with poor in-hospital outcomes after comminuted fracture surgery. Patients with any comorbidities appear to have longer LOS and a higher proportion of discharge to long-term care facilities. The analysis of comminuted fractures in children in this study may provide useful information to help first responders and medical personnel evaluate and manage comminuted fractures appropriately. A detailed longitudinal, prospective study is needed to confirm results of the present study.

## Institutional review board statement

All data were obtained through request to the Online Healthcare Cost and Utilization Project (HCUP) Central Distributor, which administers the database (certificate #HCUP-29L41EYS3). This study conforms to the NIS data-use agreement with HCUP. Because this study analyzed secondary data from the NIS database, patients and the public were not involved directly. The study protocol was submitted to the Institutional Review Board (IRB) of MacKay Memorial Hospital, which exempted the study from IRB approval. Since all data in the NIS database are de-identified, the requirement for informed consent was also waived.

## Data Availability

The original contributions presented in the study are included in the article/Supplementary Material, further inquiries can be directed to the corresponding author.
